# Gene Expression in the Physiology and Pathology of Neurons

**DOI:** 10.3390/ijms21165716

**Published:** 2020-08-10

**Authors:** Jacopo Meldolesi

**Affiliations:** San Raffaele Research Institute and Vita-Salute San Raffaele University, Department of Neuroscience, 20132 Milan, Italy; meldolesi.jacopo@hsr.it

**Keywords:** gene transcription, gene expression, neurogenesis, neuronal development, differentiation, identity, and reprogramming, axonal regeneration and guidance, tissue repair and neurogeneration, alternative splicing

## 1. Introduction

The expression of genes is the first process governing the molecular and structural specificity of the various types of cells, initiated by their transcription into the corresponding pre-mRNA. The dynamic splicing of the latter into several mRNAs makes possible the biosynthesis of multiple proteins, characterized by peculiar properties and functions. In neurons, these events are multiple and heterogeneous, Thus, neurons are the most specific and complex types of cells. Such complexity does depend not only on their marked differences with respect to non-neuronal cells, but also on the specificities acquired during differentiation. The identification and characterization of many types of neurons is attracting great interest. Their investigation is thus progressively growing, and their discussion is essential in neurobiology. Such discussion is given ample space here, dedicated to our potential readers.

The final plan of the present Special Issue includes seven reviews, followed by two articles dealing with experimental results in close areas. The contribution of our first group of reviews deals with the development of neurons in the brain. In particular, Samir Vaid and Wieland B. Huttner report on the spatio-temporal dynamics and the mechanisms involved in the development of brain cortical neurogenesis [1]. In the following review, by Gerry Melino and his colleagues Maria Victoria Niklison-Chirou, Massimiliano Agostini and Ivano Amelio, the presentation illustrates the neurogenesis in the mammalian brain, where adult neurons are integrated into neuronal circuits [2].

A following presented area deals with peculiar brain neurons, the dopaminergic neurons, well known for their small number, peculiar distribution and clinical functions. Simone Mesman and Marten P. Smidt illustrate the mechanisms by which these neurons acquire their identity and function [3], while Umberto Di Porzio and his colleagues, Floriana Volpicelli, Carla Perrone Capano, Gian Carlo Bellenchi and Luca Colucci-D’Amato, starting from the development of the same neurons, illustrate the pathogenesis of the diseases and their growing pharmacology [4].

Three additional reviews report about the dynamics of neuronal development and reprogramming processes, followed by the splicing of their transcription RNAs. The first, by Sung Wook Kim and Kyong-Tal Kim, covers one of the dynamic and functional regulated process of neurons, the guidance of axons with ensuing formation of brain circuits [5], while Raymond C.-B. Wong and his colleagues Roxanne H.-C. Liou, Thomas L. Edwards and Keith R. Martin, illustrate that, in various types of neurons, reprogramming sustains various therapeutic applications, inducing brain tissue repair and neuroregeneration [6]. The final review, by myself, deals with the process by which almost all forms of pre-RNA, spliced during or upon gene transcription, govern, from single pre-RNAs, the generation of multiple mRNAs, structurally and functionally distinct from each other [7].

The two articles included in the SI expand the relevance of two aspects already emphasized in the reviews. Linyi Chen, together with his colleagues, illustrates the role of the WNT3A gene in neuronal regeneration [8]. Such important results are connected to the neuronal reprogramming presented in the review by Wong. The second paper, by Yi Ding et al., deals with the role of a synaptic protein, synaptotagmin 1, in the generation of the neuropathic pain [9], a process relevant also in several reviews of this SI.

As a result of the choice of appropriate areas and the excellence of the author presentations, the contribution of the present SI is of potential interest for many specialists in the field. The presented processes are traditionally distinct from each other. In the present SI, however, the distinctions are not extensive. We are confident, therefore, that they will be read and widely reported by significant scientists of the neuroscientific community.
